# 3D Cancer Models: The Need for a Complex Stroma, Compartmentalization and Stiffness

**DOI:** 10.3389/fbioe.2021.660502

**Published:** 2021-04-12

**Authors:** Judith Pape, Mark Emberton, Umber Cheema

**Affiliations:** ^1^Division of Surgery and Interventional Science, Department of Targeted Intervention, Centre for 3D Models of Health and Disease, University College London, London, United Kingdom; ^2^Faculty of Medical Sciences, University College London, London, United Kingdom

**Keywords:** 3D models, tissue-engineering, tumor stroma, compartmentalization, stiffness, collagen density, stromal cells, extracellular matrix

## Abstract

The use of tissue-engineered 3D models of cancer has grown in popularity with recent advances in the field of cancer research. 3D models are inherently more biomimetic compared to 2D cell monolayers cultured on tissue-culture plastic. Nevertheless 3D models still lack the cellular and matrix complexity of native tissues. This review explores different 3D models currently used, outlining their benefits and limitations. Specifically, this review focuses on stiffness and collagen density, compartmentalization, tumor-stroma cell population and extracellular matrix composition. Furthermore, this review explores the methods utilized in different models to directly measure cancer invasion and growth. Of the models evaluated, with PDX and *in vivo* as a relative “gold standard”, tumoroids were deemed as comparable 3D cancer models with a high degree of biomimicry, in terms of stiffness, collagen density and the ability to compartmentalize the tumor and stroma. Future 3D models for different cancer types are proposed in order to improve the biomimicry of cancer models used for studying disease progression.

## Introduction

Bioengineering humanized 3D models of cancer will eventually replace the need for animal models (Kimlin et al., 2013). This is an important goal in terms of the 3R (replacement, reduction and refinement) framework to perform more humane animal research (Díaz et al., 2020). There is a growing realization that culturing cells in 2D monolayers does not truly recapitulate the immediate spatial, cellular, tensile and chemical environment of highly complex tumors and their stroma (Valkenburg et al., 2018). Increasingly, the focus has been to utilize different approaches to bioengineer 3D models to better understand tumor growth. These models offer the opportunity to not only model the cancer mass itself but also the surrounding stroma, which is important in promoting and directing cancer invasion (Weigelt et al., 2014). This review aims to outline the benefits and limitations of different 3D models currently used whilst exploring how the tumor stroma in particular can be engineered to be biomimetic and tissue specific.

### Types of 3D Cancer Models and the Tumor Stroma

**FIGURE 1:**
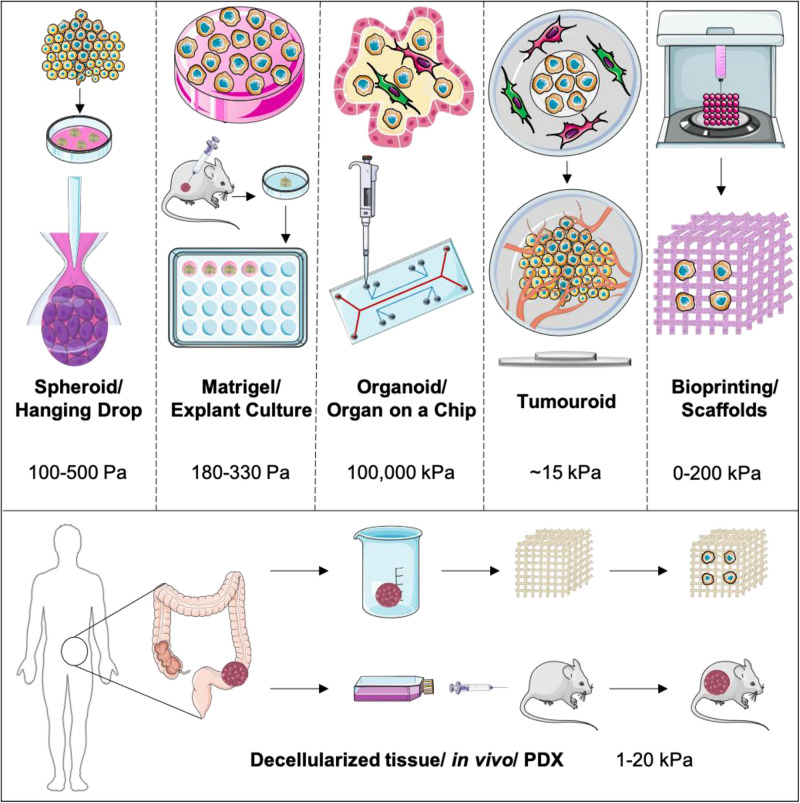
Outlining the main 3D models used currently to model cancer and the cancer stroma. These are represented with increasing biomimicry and complexity placing PDX and *in vivo* models as the golden standard.

As outlined in Figure 1, there are a number of engineering approaches taken to model cancer in 3D, with an aim to increase and accurately model the biomimetic complexity (Pampaloni et al., 2007). A critical aspect of these models is the accurate compartmentalization of both the tumor and the stroma whilst creating a boundary, allowing for cellular cross-talk and cellular migration between these compartments (Truong et al., 2016). In order to achieve distinct compartmentalization, the ECM should be able to mimic and reproduce the biomechanical properties of the native tissue or organ as this is essential for cell attachment within each specific compartment (Lam et al., 2014). The contact to non-biomimetic tissue-culture plastic needs to be eliminated and good permeability of oxygen and nutrients needs to be achieved.

### Spheroids and Hanging Drops

Spheroids are clusters of cancer cells grown on low-attachment tissue culture plates. This formation allows cancer cells to be in immediate proximity to one another in a 3D configuration as they have formed a “mass” and this provides an anchor for the accumulation of cell-generated collagen (de Angelis et al., 2018). However, within spheroid formations, significant aspects of the tumor environment are not replicated because of the lack of stromal cells and their associated affects. The main benefits of spheroids and hanging drops are their low cost, high throughput, reproducibility and ease of use (Lucendo-Villarin et al., 2020). Spheroids also exhibit oxygen gradient formation, where cell death is observed within the core where hypoxia is greatest. Whilst nutrient and oxygen permeability is high at the spheroid surface, due to direct contact with nutrient media, there is no space for the stroma compartments, and therefore limited tumor-stromal interactions other than when using a co-culture within the actual spheroid (Yakavets et al., 2019). Furthermore, there is no extracellular matrix present to mimic the biomechanical properties of native tissue, particularly those of the inherently stiff tumor tissue. The cells may still have tissue-culture plastic contact to the low-attachment plates as there is no collagen for them to attach to. The greatest limitation is that since there is no compartmentalization between cancer and stromal cells, even within a co-culture, the distance of invasion is not measurable within a spheroid. Closely related to spheroids is the hanging drop approach. This method utilizes the tensile force of a drop of growth media to force the cells to cluster together in a spheroid formation (Aijian and Garrell, 2015). This method also has similar limitations to the low-attachment plate spheroids grown.

### Matrigel^®^ and Hydrogels

As we move onto more complex 3D models, biological scaffolds such as decellularized matrices, Matrigel^®^ and collagen are used to provide an extracellular matrix (Tanner and Gottesman, 2015) for the cancer cells to populate in order to achieve biomimicry for the initial cancer mass. Next to collagen hydrogels, there are also a number a favorable biomaterials to recreate the ECM in a similar manner such as decellularized human tissue, 3D bioprinted hydrogels (Mollica et al., 2019), hyaluronan printed hydrogels that are tunable in terms of stiffness (Bonnesoeur et al., 2020), and self-assembling peptide hydrogels (Yang et al., 2020). This allows for the co-culture with stromal cells as well as incorporating chemical factors and extracellular matrix proteins (Nyga et al., 2011). Whilst these models can be streamlined to become high throughput and generally have good nutrient and oxygen permeability, they can be costly. Matrigel^®^ in particular is also known for having great batch to batch variability in terms of added ECM components. Whilst particularly primary cancer cells grow well in Matrigel^®^, this cocktail of basement membrane proteins originally extracted from the Engelbert–Holm–Swarm mouse sarcoma brings great uncertainly with it. Although there is no precise recipe for Matrigel^®^, its general composition is defined as 60% laminin, 30% collagen IV and 8% entactin. Additionally to this a number of tumor growth promoting factors such as TGF-b, EGF and VEGF are added at unknown concentrations making it a source of high variability in experimental results (Hughes et al., 2010). It is difficult to achieve distinct tumor-stroma compartments with such low matrix densities. Some might also argue what the relevance of implanting tumor cells into a basement membrane matrix would be, when human tissue consists primarily of type I collagen (von der Mark, 2006) additionally to proteoglycans, glycosaminoglycans and other important fibrillar and non-fibrillar ECM components (Theocharis et al., 2003).

### Self-Assembled Organoids, Flow and Organ-on-a-Chip

Self-assembled organoids, originally used for stem cell research, have been utilized for cancer research. Bioreactors and flow systems are used to force cells into formation (Boj et al., 2015). These allow for co-cultures to be utilized and interstitial fluid pressure conditions to be recreated. Compartmentalization is achievable in these types of models and a tumor-stroma boundary can be generated. Qin et al. (2020) within the Tape Lab at University College London have recently performed single-cell analysis on colorectal cancer organoids showing the highly sophisticated analysis tools possible on these types of models. In parallel to advanced co-culture self-assembling organoid models, microfluidic organ-on-a-chip models have been used with increasing popularity. The OrganoPlate^®^ platform proposed by Schutgens et al. (2019) within the Hans Clevers group at the Hubrecht Institute, enables up to 40 microfluidic cell culture chips to be embedded in a 384-well plate to study renal tubuloids for high-throughput personalized disease modeling. These are numbers that are simply not possible with the majority of 3D models at the moment. Whilst some might argue that tissue-culture plastic contact with the cells is not eliminated, these models have exceptionally high permeability to oxygen and nutrient flow can be regulated. In a number of organ-on-a-chip models, the distance of invasion can be measured between tumor and stromal cells. Cost can be kept relatively low and high throughput studies have been set up. Unfortunately, these models can lack ECM components unless gels such as collagen or Matrigel^®^ are injected into the channels also (Zhang et al., 2018).

### Tumoroids

There is growing research around cancer specific organoids with a dense extracellular matrix provided from type I collagen or other biomaterials. These models have been termed as “tumoroids” (Magdeldin et al., 2017). These are meant to be used as a pre step to *in vivo* work as they can recapitulate not only the cancer mass itself but also the stromal environment. The tumor boundary is accurately modeled by implanting a cancer mass within a stromal compartment. The distance of invasion can be measured directly from the origin (Pape et al., 2019). Whilst these models can be high cost depending on the tumor type, they have a high level of reproducibility and oxygen and nutrient permeability is sufficient. Within such models the diffusion coefficient of both glucose and oxygen are high (Rong et al., 2006; Cheema et al., 2012). Modeling and validation of oxygen consumption shows this measure to be cell-specific, signaling the distinct metabolic profile of different cell populations (Streeter and Cheema, 2011). The tumoroid model can be used in combination with other systems to increase stromal biomimicry. Azimi et al. (2020) within the Dwek lab at University of Westminster have successfully demonstrated that flow induces an aggressive cancer phenotype with a reduced responsiveness to doxorubicin.

### Bioprinting Scaffolds and Cellularized Bio-Inks

3D printing has gained popularity not only in biological applications within recent years. A wide range of bio-inks such as alginate and polyvinyl alcohol (PVA) have been developed that enable the printing of scaffolds to desired structures. Some of these bio-inks, such as PVA, have the ability to be highly manipulated (Luo et al., 2017). This means that the concentration and therefore porosity and stiffness can be tunable (Bonnesoeur et al., 2020). A number of these bio-inks have also shown cell viability of up to 100% when cells are seeded onto the scaffolds for up to 12 days (Mohanty et al., 2015). Other bio-inks that have cells already mixed in before printing also exist. These can be blends of agarose, gelatine and collagen (Gopinathan and Noh, 2018). Some collagen based bio-inks have shown to observe cancer cell behavior under flow conditions as demonstrated by Campos et al. (2019) from Aachen University. These applications have a number of benefits and rightly so are a fast evolving field of tissue-engineering. Some of the limitations of these models may be that due to their artificially manufactured nature and origin of bio-inks used, cell viability and attachment can be low.

### Decellularized Scaffolds, Patient Derived Xenograft (PDX), and *in vivo* Models

Decellularized scaffolds of porcine, bovine and human origin are a popular choice to re-seed with desired cell types. These decellularized scaffolds can be stripped of all animal components, avoiding the trigger of an immune reaction. Furthermore, decellularized scaffolds model the native tissue more closely than a tissue-engineered one. A limitation however is that a number of decellularization methodologies may alter the stiffness and porosity of the native tissue, which in turn will affect cellular response. Methods for decellularization include the use of harsh chemicals and enzymes, which effectively strip the cellular components within tissues, leaving behind intact matrix (Crapo et al., 2011). Some of these chemicals can be hard to remove from tissues and they either physically alter the matrix or remain in trace amounts affecting the viability of newly added cells. Recently the vacuum-assisted osmotic shock method has proven to be extremely effective at removal of cellular components whilst maintaining ECM integrity for cartilage and allowing new cell infiltration with a high degree of viability (Vas et al., 2018).

PDX models are the gold standard and a very strong tool for pre-clinical *in vivo* models of cancer especially when orthotopically transplanted (Boj et al., 2015). These models use patient-specific samples and utilize a host animal to implant tumor explants/fragments. This gives the benefit of a biomimetic, intact ECM and allows for the compartmentalization of tumor and stromal tissue. Tumor growth can be directly quantified and oxygen and nutrients are readily available through the host’s circulatory system. A major limitation of PDX models however is that the tumor microenvironment cannot be accurately recreated due to only a limited amount of stromal cells such as cancer associated fibroblasts and endothelial cells being explanted together with the tumor sample taken from the patient, which over time will be replaced by the host cells (Yada et al., 2018). The PDX model is not completely humanized, and therefore will always have the inherent issues of interspecies variability. Additionally, the adaptive immune component of cancer resistance cannot be accurately modeled, mainly due to the fact that *in vivo* models of cancer are generated in immune-deficient mice in which the inherent immune response is not fully functional (O’Brien et al., 2007). This therefore means that the immune response to cancer cells would not be the one naturally occurring in human tissue. With recent developments in 3D cancer modeling some complex models now incorporate immune cells to allow for this process (Gajewski et al., 2013). Furthermore, the use of animal models can be very high cost financially and ethically.

## Current Challenges in 3D Modeling of Cancer and the Stroma

Whilst several groups have adapted 3D models into their research, some physical and cellular microenvironmental features remain limited. These include stiffness, stromal complexity and compartmentalization. Out of the five groups of 3D models discussed within the last paragraphs, only two are able to achieve physical stiffness and a level of biomimetic ECM adequate to promote cellular growth and invasive phenotype; Matrigel^®^ and tumoroids. This leads to the fact that cancer and stromal cells will not communicate or grow in a biomimetic manner.

### Stiffness and Collagen Density

Although materials like Matrigel^®^ offer a nutrient rich tumor microenvironment, their low stiffness lies within the native stiffness range of a limited amount of human tissues, including brain tumor models. In the case of Matrigel^®^, the stiffness is 180 Pa, even lower than a collagen type I hydrogel at 330 Pa (Weigelt et al., 2014). Colon carcinoma has a stiffness ranging from 1 to 4 kPa (Brauchle et al., 2018), most muscle tissue has a range of 5–20 kPa (Gefen and Dilmoney, 2007) and femoral bone will have a stiffness range of about 15–20 GPa (Zhao et al., 2018). In comparison, tissue-culture plastic will have a stiffness of up to 100,000 kPa (Skardal et al., 2013). A major benefit of bio printable scaffolds is certainly the scope of manipulation in terms of stiffness. PVA can be used at concentrations resulting in stiffnesses from 0 to up to 200 kPa (Oh et al., 2016). Tumoroids consists of plastic-compressed collagen, which increases the collagen concentration from 0.2% by a 48-fold to ~10% (9.59 ± 1.28) (Magdeldin et al., 2017). These numbers indicate that the stiffness of tumoroids after plastic-compression would also increase by a 48-fold, leading to an approximate stiffness of ~16 kPa. Another aspect is collagen concentration within 3D models. Spheroids and hanging drop formations will have limited collagen density other than what the cells produce, critically dependent upon the media providing the correct co-factors necessary for collagen production such as ascorbic acid (Kishimoto et al., 2013). This stiffness is measurable through AFM and has been shown to be very low in the range of 100–500 Pa (Giannetti et al., 2020). Microfluidic devices will also not have any collagen unless injected, mainly hydrogels. Hydrogels have a collagen concentration of 0.2% or less, whilst Matrigel^®^ is available at 0.2–0.4%. Putting this into the perspective of the collagen concentration within native tissue, the collagen density within tendons for examples is 12% and accounts for 1–10% of skeletal muscle dry weight (Gillies and Lieber, 2011). This collagen density directly impacts the stiffness of the ECM the 3D model provides. Tumor tissue in particular will have gradients of stiffness and collagen concentration during tumor development due to the heterogenous nature of cancer cells and their adaptivity to their physical and chemical environment. Furthermore, stiffness if not only dependent on collagen itself, but rather a combination of all ECM components as a whole (Malandrino et al., 2018).

### Extracellular Matrix Components and Stromal Populations

Looking at other ECM components essential for cell growth, this category is often neglected within 3D set-ups. A number of cancer types require laminins and fibronectin for growth as well as additional collagen subtypes. Specifically in breast cancers, the presence of factors such as fibronectin can drive a more aggressive breast cancer type (Nolan et al., 2020). Taking a further look at the stromal cell population, numerous groups have made the effort to include fibroblasts or the highly differentiated cancer associated fibroblasts (CAFs) (Pape et al., 2020) and even endothelial cells in order to form primitive vascular networks in the presence of laminin (Stamati et al., 2014). This aids the cellular cross-talk between cancer and stromal cells generating gradients of angiogenic and fibrotic factors. Studying vascular network formation, remodeling and disruption in relation to a growing tumor mass is important in order to understand the cancer’s ability for nutrient acquisition and metastasis (Perea Paizal et al., 2021) in addition to identify future drug targets (Farnsworth et al., 2014). In terms of studying the reactive cancer environment, the biggest gap in modeling the cancer stroma consists of not including the appropriate immune cell population. Modeling the sequence of immune events is tricky, especially in 3D. Many epithelial cancers will have populations of inherent macrophages resident in tissues and T-cells and NK-cells will be recruited as the immune reaction kicks off (Gonzalez et al., 2018). In some cancers this immune reaction will be severe such as in malignant gliomas (Liu et al., 2003) and prostate cancer (Vitkin et al., 2019), whilst in others like osteosarcoma the inherent immune response may not be as strong (Heymann et al., 2019). Another factor that is understudied is the inclusion of neuronal networks, within the stromal compartment of 3D models, critical for modeling neuronal and neuronal-glial tumors of the central nervous system (Rampazzo et al., 2013).

### Compartmentalization Between Cancer and Stroma

Another literal gap within a number of 3D models of cancer is the compartmentalization between cancer mass and stroma. The interaction of cancer and stromal cells has had a lot of attention within recent years, however, often these cells will be mixed together in co-cultures, and expected to self-aggregate (Rowe and Weiss, 2009). Some attempts have been made with a number of invasion assays to see how cancer cells cross the barrier and interact with the stromal population (Yu and Machesky, 2012). This barrier is not often physical and rarely in the context of a stiff collagen ECM. Cancer cells grow together in clusters until a certain size is reached (Pape et al., 2019). Furthermore, some tumors are actually made up in large by the bulk stroma mass, such as in the case of pancreatic cancer (Gore and Korc, 2014). It is not appropriate to model cancers without this physical, chemical and cell barrier. The basement membrane plays a key role in a number of invasive cancers, since it often signifies the staging and aggressiveness of the cancer (Chang and Chaudhuri, 2019). It is therefore important to model this barrier between one compartment, organ or tissue to another. This will allow for the measurement of cancer growth and invasion. Human tissue is defined by barriers and compartments (organs). Furthermore, a number of cancers arise at these borders where cells change from one type to another or tissue environments change. Some examples would be the transition zones from colon to rectum, stomach to esophagus and the end of the cervix (De Wever and Mareel, 2003).

## Discussion and Proposed Future Models

**FIGURE 2:**
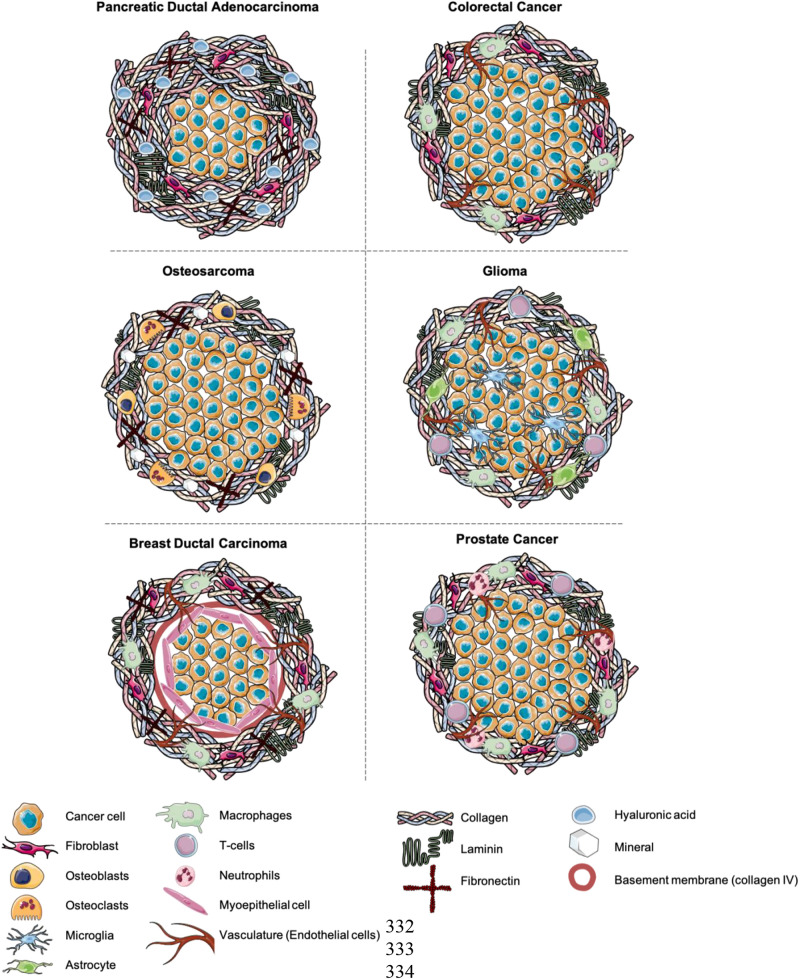
Suggestions for the future modeling of 3D cancer models. Composed of a central cancer mass and stromal compartment containing stromal cell populations and ECM components.

Future developments within the field require a shift toward modeling the extracellular matrix in a physiologically relevant manner. Furthermore, more attention needs to be paid toward modeling the tumor stroma (Veenstra et al., 2018). Tumors need to be grown in a physiologically relevant environment receiving the same cues as they would in the native tissue. In Figure 2, we have outlined proposed set-ups that could be used as a guideline. It will be important to not only implant the cancer cells into a physiologically relevant, “dense” ECM, but to also model the tumor-stroma in a biomimetic manner (Barcus et al., 2013). Tumoroid models provide a platform to engineer matrix relevant models, where the density and composition can be controlled. The ability to engineer separate cancer masses and stromal compartments, and then bring them together, more closely mimics the natural growth of cancer. It is critical to engineer biomimetic stromal compartments, containing appropriate cell populations, including fibroblasts, endothelial cells and immune cells additionally to ECM components (Rowe and Weiss, 2009).

It is crucial that all 3D models have the scope to include patient samples. This has already been done in the past in the form of patient CAF samples within tumoroids (Pape et al., 2020). As primary cancer cells are difficult to grow in culture, this will need to be explored further. This could then lead to the utilization of personalized drug-screening platforms. Within the current climate, a lot of models not only focus on what makes tumor cells grow the fastest, but have moved to interrogate invasion, migration, cancer stem cell’s plasticity and cancer cell dormancy. This is an extremely promising outlook for the future of 3D cancer research. This can lead to more biomimetic 3D models being used to accurately model the interaction between cancer and stromal cells. Furthermore, a physiologically relevant ECM in terms of composition and stiffness will allow for the creation of barriers between the cancer mass and surrounding stroma being tissue-engineered. With the increased uptake of 3D modeling as a pre-clinical tool, the number of animal studies may be re-evaluated and lead to a more ethical approach to research.

## Author Contributions

JP, ME, and UC conceptualized and planned the manuscript. JP created the figures and wrote the manuscript. ME and UC edited the manuscript. All authors have approved the manuscript.

## Conflict of Interest

The authors declare that the research was conducted in the absence of any commercial or financial relationships that could be construed as a potential conflict of interest.
